# The Invasive Plant *Amaranthus spinosus* L. Exhibits a Stronger Resistance to Drought than the Native Plant *A. tricolor* L. under Co-Cultivation Conditions When Treated with Light Drought

**DOI:** 10.3390/plants13162251

**Published:** 2024-08-13

**Authors:** Congyan Wang, Yingsheng Liu, Chuang Li, Yue Li, Daolin Du

**Affiliations:** 1 School of Environment and Safety Engineering, Jiangsu University, Zhenjiang 212013, China; 3202202017@stmail.ujs.edu.cn (Y.L.); 2222209081@stmail.ujs.edu.cn (C.L.); 2222209050@stmail.ujs.edu.cn (Y.L.); 2 Jiangsu Collaborative Innovation Center of Technology and Material of Water Treatment, Suzhou University of Science and Technology, Suzhou 215009, China; 3 Key Laboratory of Forest Plant Ecology, Ministry of Education, Northeast Forestry University, Harbin 150040, China; 4 Jingjiang College, Jiangsu University, Zhenjiang 212013, China

**Keywords:** biomass, competitive advantage, external stress, invasive plants, stress resistance index

## Abstract

Drought may facilitate the invasion process of invasive plants, mainly because invasive plants can obtain a stronger growth competitiveness than native plants under drought. It is therefore imperative to illuminate the mechanisms underlying the successful invasion of invasive plants under drought, with a particular focus on the differences in the resistance of invasive and native plants to drought. This study aimed to elucidate the differences in the resistance between the invasive plant *Amaranthus spinosus* L. and the native plant *A. tricolor* L. to drought under a gradient of drought. The resistance of co-cultivated *A. spinosus* to drought was significantly higher than that of co-cultivated *A. tricolor* under light drought. Hence, *A. spinosus* may obtain a stronger competitive advantage than *A. spinosus* under co-cultivation conditions when treated with light drought. The resistance of the two plants to drought may be predominantly influenced by their height and biomass. This present study also defines a method for evaluating the stress resistance of a given plant species to stress by calculating the stress resistance index. This present study offers a robust theoretical foundation for determining the stress resistance of a given plant species and the environmental management of *A. spinosus* under drought.

## 1. Introduction

All organisms are situated within a particular ecological environment. It is important to note, however, that the ecological environment in which organisms reside is not always homeostatic. During the growth process, the survival of organisms may be threatened. In particular, the ecosystems in which organisms reside are often subject to a certain degree of external stress, including, but not limited to, drought, fires, and high temperatures [1,2,3,4]. Such conditions may exert a certain influence on the growth and metabolic processes of the organisms in question [5,6,7,8]. It is evident that organisms possess the capacity to resist external stresses to a certain extent, thereby enabling them to survive within the ecological environment and ultimately prevail in the struggle for survival. In particular, the stronger resistance of organisms to external stresses may confer upon them a competitive advantage, particularly in the expansion of their ecological niche within a given habitat, specifically when subjected to stronger selective pressure mediated by those external stresses. More importantly, a stronger resistance to external stresses can promote the maintenance of a relatively stable state, thereby facilitating adaptation to surrounding habitats.

It is of greater consequence that organisms, and in particular plant species, are indispensable for the functioning of ecosystems [9,10,11,12]. It is of paramount importance to recognize that the resistance of each plant species to external stresses may fluctuate due to the variations in the species identity and/or the severity of external stresses. It is unfortunate that, at present, there is no method for quantifying the resistance of a given plant species to external stresses.

Currently, one of the most significant global environmental concerns is the advanced increase in climate change, largely due to the ongoing rise in global temperatures and the gradual onset of annual rainfall variability [13,14,15,16]. Presently, the area of the world’s land surface covered by arid and semi-arid districts is ~3.83 × 10^7^ km^2^, representing ~40% of the total land surface area of the planet [17,18]. Furthermore, it is anticipated that drought events will become more frequent and severe in the future, particularly in arid and semi-arid regions. The extent of these regions is also expected to continue to expand due to the increasing frequency and intensity of climate change and anthropogenic activities [14,15,16,17]. It is also important to note that drought can facilitate the invasion process of invasive plants (IPSs), as IPSs can often outcompete native plants in the context of drought [5,6,8,19]. However, IPSs can exert a considerable influence on native ecosystems. In particular, IPS can disrupt the structure and ecological function of native ecosystems, which can result in the loss of native biodiversity [7,20,21,22]. It is therefore imperative to elucidate the mechanisms underlying the successful invasion of IPSs in the context of drought, with a particular focus on the differences in the resistance of IPSs and native plants to drought. Nevertheless, the advancement of research concerning the discrepancy in the resistance between invasive and native plants to external stresses, particularly in relation to a spectrum of drought conditions, is currently limited.

The aim of this present study was to propose a methodology for quantifying the resistance of a given plant species to external stresses. A case study was conducted to ascertain the discrepancies in the resistance between the invasive plant *Amaranthus spinosus* L. and the native plant *A. tricolor* L. to drought under a gradient of drought (including the control, a light drought, and a heavy drought). The two plants are both herbaceous species of *Amaranthus*, belonging to the Amaranthaceae family. At the family level, Amaranthaceae was found to have the fourth highest species number of IPSs (25 species), following the families of Asteraceae (49 species), Fabaceae (35 species), and Poaceae (28 species) in East China [23]. At the generic level, the species number of IPSs included in *Amaranthus* (with 16 species, representing ~5.35% of the total species number of IPSs in East China) is significantly higher than that included in other genera in East China (Yan et al., 2021). Furthermore, *A. spinosus* originated in North America, and the species number of IPSs in China that originated in North America is significantly higher than that of species originating in other countries and/or regions [23,24,25,26]. It is of particular importance to note that *A. spinosus* is currently registered as one of the most notorious IPSs in China, largely due to the significant threats it poses to the structure and ecological function of native ecosystems. Furthermore, *A. spinosus* and *A. tricolor* can co-occur in the same community, including wastelands, farmland edges, and areas near major roads in southern Jiangsu, China. The areas in China where the two plants have been distributed are also among the areas most severely affected by drought [15,16,17].

The objective of this present study was to propose a question: Does the invasive plant *A. spinosus* exhibit a greater resistance to drought than the native plant *A. tricolor* under a gradient of drought? In addition, this present study also aims to define a methodology for quantifying the stress resistance of a given plant species to external stresses by calculating the stress resistance index.

## 2. Materials and Methods

### 2.1. Experimental Design

The seeds of *A. spinosus* were collected in October 2019 from Zhenjiang, Jiangsu, China (32.11° N, 119.53° E). The selected ecosystems were classified as wastelands. *Amaranthus spinosus* was the sole species of IPS present in the sampled communities. It is plausible that the sampled *A. spinosus* individuals may have dispersed naturally within the sampled communities. Zhenjiang is located within a north subtropical monsoon humid climate zone. The annual mean temperature is ~17.0 °C, with a monthly mean temperature reaching a maximum of ~30.5 °C in August and a minimum of ~3.1 °C in December. The annual precipitation is ~919.5 mm, with the monthly mean precipitation reaching a maximum of ~208.7 mm in July and a minimum of ~9.2 mm in May. The annual sunshine duration is ~2014.0 h, with a monthly mean sunshine time reaching a maximum of ~218.1 h in July and a minimum of ~101.5 h in November [27]. The seeds of *A. tricolor* cv. *xinbai* were procured from Qingxianchunfeng Vegetable Cultivars Breeding Base, Cangzhou, China, at a local vegetable market.

**Figure 1:**
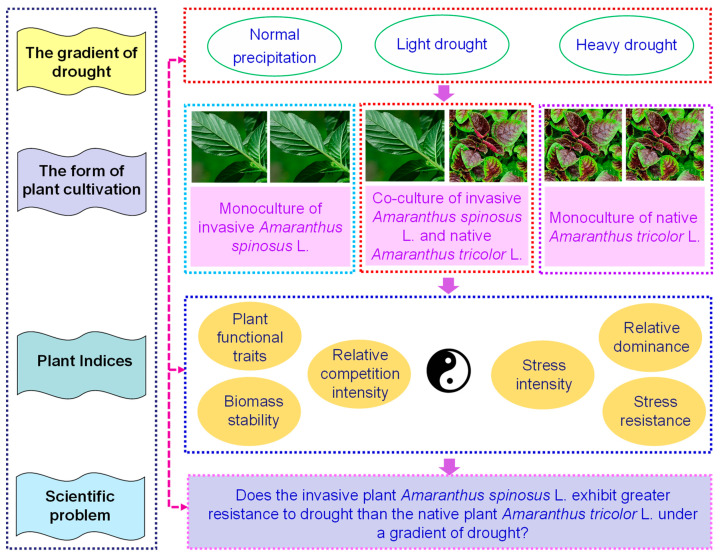
The chart of the experiment design of this present study.

A pot competitive co-culture experiment was conducted to assess the growth of the two plants. This experiment was conducted in June 2020. Seeds of the two plants were sown in garden pots (manufacturer: Huazhihuijing Co. Ltd., Suqian, Jiangsu, China; diameter: ~30 cm; height: ~17.2 cm). Six uniform, robust seedlings of *A. spinosus* and/or *A. tricolor* were planted per garden pot. The pasture yellow soil (manufacturer: Zhongfangnongmu Co. Ltd., Taizhou, Jiangsu, China; pH value: ~6.5; organic content: ≥40%; ~5 kg per garden pot) was selected as the growth substratum. The rationale for utilizing pasture yellow soil as the growth substratum was to eliminate the possibility of IPSs becoming established in soil obtained from a natural field. Three distinct forms of plant cultivation were employed: (1) six *A. spinosus* seedlings were planted per garden pot in the monoculture; (2) three *A. spinosus* seedlings and three *A. tricolor* seedlings were planted per garden pot in the co-culture; and (3) six *A. tricolor* seedlings were planted per garden pot in the monoculture. All garden pots were treated with a gradient of artificially simulated drought through the addition of varying quantities of water; specifically, this included (1) the control (100% of the normal monthly mean precipitation of June of Zhenjiang, which equates to ≈430 mL/d per garden pot); (2) a light drought (60% of the normal monthly mean precipitation of June of Zhenjiang, which equates to ≈258 mL/d per garden pot); and (3) a heavy drought (20% of the normal monthly mean precipitation of June of Zhenjiang, which equates to ≈86 mL/d per garden pot). In this present study, the gradient of artificially simulated drought was identified in accordance with the Chinese industry standards (No.: GB/T 20481; name: Classification of Meteorological Drought of China) [28]. This present study employed a range of plant cultivation combinations (i.e., monocultural *A. spinosus*, co-cultivated *A. spinosus* and *A. tricolor*, and monocultural *A. tricolor*) and a gradient of drought combinations (i.e., the control, a light drought, and a heavy drought). Three replicates were conducted for each treatment. The experimental design of the present study is illustrated in Figure 1.

Seedlings of the two plants were cultivated in an artificial greenhouse at Jiangsu University, Zhenjiang (32.2061° N, 119.5128° E), under conditions of natural sunlight.

Upon completion of the pot competitive co-culture experiment, all individuals of the two plants were used for analysis. This included the determination of the values of the plants’ functional traits, the biomass stability index, the levels of osmotic adjustment and the activities of antioxidant enzymes, the stress resistance index of the two plants to drought, the relative competition intensity and relative dominance indices of *A. spinosus*, and the stress intensity index of drought on the growth of the two plants.

In particular, the analyzing method and the values of the plants’ functional traits (including height, ground diameter, fresh and dry weights, water content, leaf length and width, specific leaf area, leaf fresh and dry weight weights, leaf water content, and leaf chlorophyll and nitrogen contents), the biomass stability index, the levels of osmotic adjustment (including malondialdehyde and proline contents) and the activities of antioxidant enzymes (including catalase, peroxidase, and superoxide dismutase activities) of the two plants, the relative competition intensity and the relative dominance indices of *A. spinosus*, and the stress intensity index of drought on the growth of the two plants have been previously described in a related study [6].

### 2.2. Method for Determining the Stress Resistance Index

To assess the degree of resistance exhibited by a given plant species to stress (which in this study is also referred to as drought), the stress resistance index (*SRI*) was calculated according to the following formula:

$$SRI=1-\frac{B_{ns}-{\;B}_{s}}{B_{ns}}$$

where *B_s_* and *B_ns_* represent the total biomass of the plant species *i* under the condition with stress and the condition without stress, respectively. In particular, plant species with a higher value of *SRI* demonstrate a greater degree of stress resistance compared to those with a lower value of *SRI*.

### 2.3. Statistical Analysis

Shapiro–Wilk’s test and Bartlett’s test were conducted to determine the extent of departure from the normality and the homogeneity of the examined variances, respectively. A multiple comparison with the Tukey’s test was employed to assess the statistical differences in the values of the stress resistance index of the two plants across different treatments. Correlation patterns between the values of the plants’ functional traits, the levels of osmotic adjustment and the activities of antioxidant enzymes, and the stress resistance index of the two plants were assessed by using correlation analysis and principal component analysis (PCA), respectively. *p* ≤ 0.05 was considered to signify a statistically significant difference. Statistical analyses were conducted using IBM SPSS Statistics 26.0 (IBM, Inc., Armonk, NY, USA).

## 3. Results and Discussion

**Figure 2:**
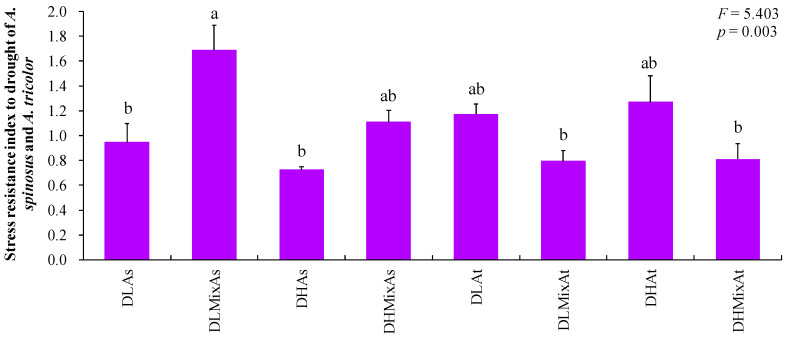
Differences in the values of the stress resistance index of *A. spinosus* and *A. tricolor* among different treatments. Bars (mean and standard error, *n* = 3) with different lowercase letters represent statistically significant differences at 0.05 probability. Abbreviations: DLAs, monocultural *A. spinosus* treated with a light drought; DLMixAs, co-cultivated *A. spinosus* treated with a light drought; DHAs, monocultural *A. spinosus* treated with a heavy drought; DHMixAs, co-cultivated *A. spinosus* treated with a heavy drought; DLAt, monocultural *A. tricolor* treated with a light drought; DLMixAt, co-cultivated *A. tricolor* treated with a light drought; DHAt, monocultural *A. tricolor* treated with a heavy drought; DHMixAt, co-cultivated *A. tricolor* treated with a heavy drought.

The values of the stress resistance index of co-cultivated *A. spinosus* treated with a light drought were found to be significantly higher than that of monocultural *A. spinosus* treated with a light drought (*p* < 0.05; Figure 2). However, no significant differences were observed in the values of the stress resistance index between monocultural *A. spinosus* treated with a heavy drought and co-cultivated *A. spinosus* treated with a heavy drought (*p* > 0.05; Figure 2). Thus, the resistance of co-cultivated *A. spinosus* to drought was found to be significantly higher than that of monocultural *A. spinosus* under a condition with a light drought. This may be attributed to the higher biomass stability index of co-cultivated *A. spinosus* and the lower stress intensity index of drought for the growth of co-cultivated *A. spinosus* compared to monocultural *A. spinosus* when treated with a light drought [6]. It can therefore be concluded that *A. spinosus* displays a more pronounced competitive advantage under the co-cultivation conditions compared to the monoculture condition under a condition with a light drought. Nevertheless, no significant differences were observed in the stress resistance index between monocultural *A. tricolor* and co-cultivated *A. tricolor* regardless of the gradient of drought (*p* > 0.05; Figure 2). Thus, the form of plant cultivation did not have a significant impact on the resistance of *A. tricolor* to drought.

More importantly, the stress resistance index of co-cultivated *A. spinosus* treated with a light drought was found to be significantly higher than that of co-cultivated *A. tricolor* treated with a light drought (*p* < 0.05; Figure 2). However, no significant differences were observed in the stress resistance index between co-cultivated *A. spinosus* treated with a heavy drought and co-cultivated *A. tricolor* treated with a heavy drought (*p* > 0.05; Figure 2). It was thus demonstrated that the resistance of co-cultivated *A. spinosus* to drought was significantly higher than that of co-cultivated *A. tricolor* under the condition of light drought. This phenomenon may be attributed to the higher biomass stability index of co-cultivated *A. spinosus* and the lower stress intensity index of drought for the growth of co-cultivated *A. spinosus* compared to co-cultivated *A. tricolor* when treated with a light drought [6]. An additional potential explanation for this phenomenon is that co-cultivated *A. spinosus* exhibits a greater height and leaf chlorophyll and nitrogen contents compared to co-cultivated *A. tricolor* when treated with a light drought [6]. The greater height and leaf chlorophyll and nitrogen contents of co-cultivated *A. spinosus* may confer a competitive advantage in light acquisition and its absorption and utilization efficiency compared to co-cultivated *A. tricolor* under the condition of light drought. In general, light is a vital component of the growth process for plants [29,30,31,32]. It was thus demonstrated that co-cultivated *A. spinosus* displayed markedly greater resistance to drought in comparison to co-cultivated *A. tricolor* under the condition of light drought. It can thus be proposed that *A. spinosus* may exhibit a more pronounced competitive advantage than *A. tricolor* under co-cultivations condition when treated with a light drought.

**Figure 3:**
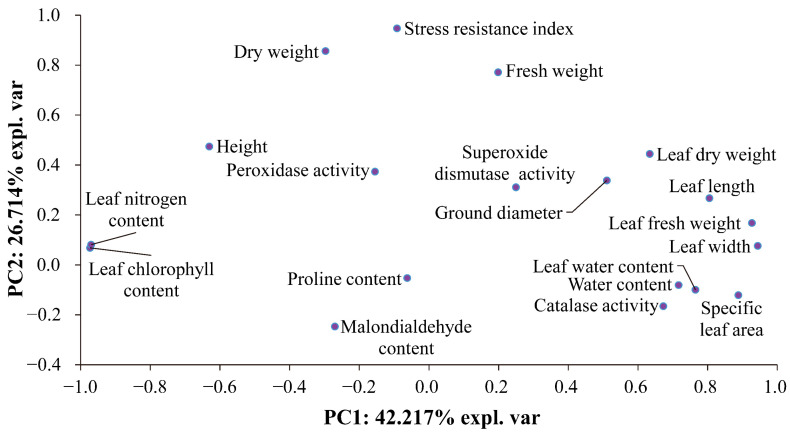
PCA of the correlation patterns of the values of the plants’ functional traits, the levels of osmotic adjustment and the activities of antioxidant enzymes, and the stress resistance index of *A. spinosus* and *A. tricolor*. The *X*-axis and the *Y*-axis account for 42.217% and 26.714% of the total variation, respectively.

The results of the correlation analysis (*p* < 0.01; Table 1) and principal component analysis (Figure 3) indicate that the stress resistance index of the two plants may be predominantly influenced by their height and biomass. This can be attributed to the fact that height and biomass represent a key factor in determining the competitive capacity for light acquisition and the growth competitiveness of plants [33,34,35,36]. However, the competitive capacity for light acquisition and the growth competitiveness of plants are essential for plant fitness [37,38,39,40]. Consequently, the competitive capacity for light acquisition and their growth competitiveness represent pivotal elements influencing their stress tolerance.

It is therefore of the utmost importance to halt or even stop the invasive process of *A. spinosus*, particularly under co-cultivation conditions when treated with drought, especially in the case of a light drought. The findings of this study also provide a substantial practical basis for the environmental management of *A. tricolor*, including the implementation of effective early-warning prevention and control measures, particularly in the context of a gradient of drought. It is of particular importance to modify the arid conditions in areas invaded by *A. tricolor*. This is to diminish the competitive advantage of *A. tricolor*, particularly in regard to its stress resistance, especially under the condition of a light drought.

In conclusion, this present study proposes a methodology for evaluating the stress resistance of a given plant species to stress by calculating the stress resistance index. This present study provides a robust theoretical basis for determining the stress resistance of a given plant species to stress. Moreover, this study offers a robust theoretical foundation for the environmental management of *A. tricolor* under drought.

## Figures and Tables

**Table 1:** Correlations (*r*) between the stress resistance index of *A. spinosus* and *A. tricolor* and the values of the plants’ functional traits, and the levels of osmotic adjustment and the activities of antioxidant enzymes in *A. spinosus* and *A. tricolor* (*n* = 3).

		Stress Resistance Index
Height	*r*	0.534 **
	*p*	**0.007**
Ground diameter	*r*	0.347
	*p*	0.097
Fresh weight	*r*	0.769 ***
	*p*	**<0.0001**
Dry weight	*r*	0.892 ***
	*p*	**<0.0001**
Water content	*r*	−0.146
	*p*	0.495
Leaf length	*r*	0.266
	*p*	0.210
Leaf width	*r*	0.019
	*p*	0.929
Specific leaf area	*r*	−0.201
	*p*	0.347
Leaf fresh weight	*r*	0.029
	*p*	0.893
Leaf dry weight	*r*	0.210
	*p*	0.324
Leaf water content	*r*	−0.076
	*p*	0.726
Leaf chlorophyll content	*r*	0.121
	*p*	0.573
Leaf nitrogen content	*r*	0.134
	*p*	0.533
Malondialdehyde content	*r*	−0.309
	*p*	0.142
Proline content	*r*	−0.123
	*p*	0.567
Catalase activity	*r*	−0.160
	*p*	0.454
Peroxidase activity	*r*	0.410 *
	*p*	**0.047**
Superoxide dismutase activity	*r*	0.315
	*p*	0.133

*, ** and *** indicate statistically significant differences at the 0.05, 0.01, and 0.001 probability levels, respectively. *p* ≤ 0.05 is shown in bold.

## Data Availability

No new data were created or analyzed in this study. Data sharing is not applicable to this article.

## Abbreviations

IPS	Invasive plants
*SRI*	Stress resistance index
PCA	Principal component analysis
DLAs	Monocultural *A. spinosus* treated with a light drought
DLMixAs	Co-cultivated *A. spinosus* treated with a light drought
DHAs	Monocultural *A. spinosus* treated with a heavy drought
DHMixAs	Co-cultivated *A. spinosus* treated with a heavy drought
DLAt	Monocultural *A. tricolor* treated with a light drought
DLMixAt	Co-cultivated *A. tricolor* treated with a light drought
DHAt	Monocultural *A. tricolor* treated with a heavy drought
DHMixAt	Co-cultivated *A. tricolor* treated with a heavy drought
